# Knockdown of STIL suppresses the progression of gastric cancer by down‐regulating the IGF‐1/PI3K/AKT pathway

**DOI:** 10.1111/jcmm.14440

**Published:** 2019-06-11

**Authors:** Ju Wang, Yong Zhang, Zhongxia Dou, Hongwei Jiang, Yongqiang Wang, Xiaoping Gao, Xiangyang Xin

**Affiliations:** ^1^ Department of Gastrointestinal Surgery Inner Mongolia People's Hospital Hohhot China; ^2^ Lung Cancer Centre, State Key Laboratory of Biotherapy and Cancer Centre West China Hospital, Sichuan University Chengdu China; ^3^ Department of Ophthalmology Inner Mongolia Baogang Hospital Baotou China

**Keywords:** apoptosis, cell cycle, gastric carcinoma, PI3K/AKT pathway, STIL

## Abstract

SCL/TAL1 interrupting locus (STIL) regulates the mitotic centrosome to promote the centriolar replication and cell cycling, and is associated with malignancies. However, the role and mechanism of STIL in gastric cancer (GC) remain elusive. STIL expression in GC tissue microarray was detected by immunohistochemistry (IHC). GC cells were transduced with control lentivirus or lentivirus for expression STIL‐specific shRNA and the effect of STIL silencing on the malignant behaviors of GC cells was measured in vitro and in vivo. The potential mechanisms underlying the action of STIL were analyzed by transcriptome microarray and bioinformatics. STIL expression was up‐regulated in GC tissues both in our cohort and the data from the cancer genome atlas, and positively associated with T stage and poor overall survival of GC patients. Knockdown of STIL significantly inhibited the proliferation and clonogenicity of human GC cells and attenuated the growth of implanted GC in vivo. Furthermore, STIL silencing induced cell cycle arrest in G2/M phase and apoptosis of GC cells. Transcriptome analysis indicated that STIL silencing modulated many gene expression, particularly for down‐regulating the IGF‐1/PI3K/AKT pathway. In addition, treatment with SC79, an AKT activator, significantly mitigated the effect of STIL‐silencing in GC cells. In conclusion, STIL promotes gastric carcinogenesis and progression by enhancing the IGF‐1/PI3K/AKT signaling, and STIL may be a novel target for intervention of GC.

## 1 INTRODUCTION

Gastric cancer (GC) is the fourth and fifth most common cancer in men and women, Respectively.^1^ Although the incidence and mortality of GC are decreasing dramatically over the past 50 years in developed countries, there are still 679 100 new cases occurred and 498 000 died from GC in 2015, remaining the second most common cancer and the cancer‐related death in China.^2^ Patients with early stage of GC usually have no specific symptoms and most patients with GC are diagnosed at advanced stage although great advances in imaging modalities for diagnosis of GC. Therefore, discovery of novel biomarkers for GC will be of great importance for the diagnosis and prognosis of GC.

Chromosome instability, caused by ectopic centriolar amplification, is ubiquitous in human malignancies, and occurs approximately in 50% of GC.^3^ SCL/TAL1 interrupting locus (STIL) is a critical regulator of mitotic centrosome to promote the centriolar replication and cell cycling.^4^ During the process of cell cycling, STIL expression is induced in an immediate early fashion, reaches the peak levels in mitosis and is degraded after cell cycle exits from mitosis.^5^ STIL through its coiled‐coil and STAN domains can interact with Polo‐like kinase 4, cyclin dependent kinase 1 (CDK1) and SAS‐6 to promote centriole duplication and maintain chromosome stability.^6^ It is notable that STIL expression is elevated in multiple types of cancers, such as lung cancer and pancreatic cancer and correlated with the expression of several checkpoint genes and mitotic indicators.^7^, ^8^ Furthermore, STIL has been reported to be one of the up‐regulated 17 genes in primary adenocarcinoma and their elevated expression is associated with metastasis.^9^ Hence, STIL acts as an oncogenic factor to promote the progression of several types of cancers. However, the role of STIL in the development and progression of GC has yet been explored. We aimed to examine the expression of STIL in clinical specimens and human GC cells, investigate the impact of STIL silencing on the malignant behaviors of GC cells in vitro and in vivo, and explore the potential mechanisms underlying the action of STIL in gastric carcinogenesis and progression.

## 2 MATERIALS AND METHODS

### 2.1 Tissue microarray and immunohistochemistry

The Tissue microarray (TMA), including 100 GC and 80 para‐tumor tissues, was purchased from Shanghai Outdo Biotech (Catalog no. HStmA180Su08). The samples were collected from July 2006 to April 2007. The patients were followed‐up until July 2015, with the duration from 8.2 to 9 years. Written informed consent was obtained from each patient, and the protocol was approved by the Ethics Committee of the National Engineering Center for Biochip at Shanghai. Additionally, this study was also approved by the Ethics Committee of Inner Mongolia Hospital. The TMA sections (3 µm) were de‐paraffinized and rehydrated, followed by antigen retrieval in citrate buffer in a pressure cooker. The TMA sections were treated with 3% hydrogen peroxide for 10 minutes to block the endogenous peroxidase activity and 3% bovine serum albumin. Subsequently, the sections were probed with polyclonal anti‐STIL (Abcam, USA, 1:100) at 4°C overnight. The bound antibodies were detected with horseradish peroxidase (HRP)‐conjugated second antibodies and visualized using the chromogen of diaminobenzidine, followed by counterstained with hematoxylin.

The intensity of anti‐STIL staining was scored by two pathologists using the Image‐Pro Plus 6.0 software in a blinded manner. The scores were determined based on the percentages of positive area (a) and staining density (b) and the values of staining index (a × b) were calculated. The samples were stratified by the median value of staining index for high or low STIL expression.

### 2.2 TCGA dataset analysis

gastric cancer mRNA data were downloaded from the TCGA database. A total of 402 files, containing 375 tumor and 32 non‐tumor tissues, were available for analysis. We employed the R package of “Edger” to screen differentially expressed genes (DEGs) and analyzed the levels of STIL expression in GC and matched non‐tumor tissues.

### 2.3 Cell culture and lentivirus transfection

Human GC SGC‐7901, MKN‐45, MGC80‐3 and BGC‐823 cells were purchased from Type Culture Collection of the Chinese Academy of Sciences, Shanghai. The cells were cultured in RPMI 1640 medium supplemented with 10% fetal bovine serum in a humidified atmosphere of 5% CO_2_ at 37°C. SGC‐7901 and BGC‐823 cells at 50% of confluency were transduced with control lentivirus for expressing scramble sequences (shCon), or lentivirus for expressing STIL‐specific shRNA (shSTIL, Shanghai Gene, China) at a multiplicity of infection of 10 and treated with puromycin (3 µg/mL) to establish stably STIL silencing cells. The specific sequence of shSTIL was 5'‐GTCTGGAATTACACATATCTA‐3'.

### 2.4 Quantitative reverse PCR

Total RNA was extracted from individual cell lines using HP Total RNA Kit (OMEGA BIO‐TEK, USA) according to the manufacture's protocol. Individual RNA samples (2 μg/each) were reversely transcribed into cDNA using All‐in‐One^TM^ First‐Strand cDNA Synthesis Kit (GeneCopoeiaTM). The relative levels of STIL mRNA transcripts to the control GAPDH were determined by quantitative RT‐PCR using All‐in‐One^TM^ qPCR Mix (GeneCopoeia^TM^) and the specific primers. The sequences of primers were forward, 5'‐CCCAACGCCAACTGGAGATTT‐3' and reverse 5'‐AGTCGGATGGTCTTCTCAGTC‐3' for STIL (87 bp); forward '5‐TGACTTCAACAGCGACACCCA‐3' and reverse 5'‐CACCCTGTTGCTGTAGCCAAA‐3' for GAPDH (121 bp). The data were normalized to the control and analyzed by 2^−ΔΔCt^.

### 2.5 Western blotting

The cells were harvested and lyzed in RIPA buffer, followed by centrifugation. The concentrations of total proteins in the cell lysates were determined by BCA assay. Individual cell lysates (50 µg/lane) were separated by sodium dodecyl sulfate polyacrylamide gel electrophoresis (SDS‐PAGE) on 8% and 20% gels and transferred electronically onto polyvinylidene difluoride (Millipore, USA). The membranes were treated with 5% fat‐free dry milk in TBST buffer and incubated with antibodies against STIL, PI3K, AKT, p‐AKT (Abcam, USA, 1:3000) and GAPDH (Santa‐Cruz Biotech, USA, 1:3000) at 4°C overnight. The bound antibodies were detected with HRP*‐*conjugated secondary antibodies and visualized using Immobilon^TM^ western chemiluminescent HRP Substrate (Millipore, USA).

### 2.6 Proliferation assay

The impact of STIL silencing on the proliferation of SGC‐7901 and BGC‐823 cells was determined by CCK‐8 assay. Briefly, SGC‐7901/shCon, SGC‐7901/shSTIL, BGC‐823/shCon and BGC‐823/shSTIL cells (2 × 10^3^ cells/well) were cultured in triplicate in 96‐well plates for 5 days. Every day after culture, individual wells of cells were added with 10 μL of CCK‐8 solution and cultured for another 4 hours, and measured for the absorbance at 450 nm in a microplate reader.

### 2.7 Colony formation assay

SGC‐7901/shCon, SGC‐7901/shSTIL, BGC‐823/shCon and BGC‐823/shSTIL cells (2 × 10^3^ cells/well) were cultured in triplicate in 6‐well plates for 10 days and their medium were changed every 3 days. The generated cell clones were fixed in 3.7% formaldehyde and stained with 0.25% of crystal violet (AMRESCO). The colony‐forming efficiency (CFE %) in individual wells was calculated for the ratio of the clone numbers to the cell numbers inoculated (2000 cells/well).

### 2.8 Flow cytometry for cell cycle and apoptosis

After growing at 37°C for 5 days, SGC‐7901/shCon, SGC‐7901/shSTIL, BGC‐823/shCon and BGC‐823/shSTIL cells were harvested and fixed in 70% of ethanol at 4°C overnight. The cells were digested with RNAse A and stained with propidium iodide (PI, Sigma‐Aldrich). The distribution of different phases of cells was determined by flow cytometry in a FACS Calibur (BD Biosciences). To detect spontaneous apoptosis, the different groups of cells were harvested and stained with Annexin V‐APC. The percentages of apoptotic cells were determined by flow cytometry.

### 2.9 Xenografted tumor model

The experimental protocol was approved by the Ethics Committee of Inner Mongolia Hospital. Male BALB/c nude mice (4‐5 weeks old) were purchased from Shanghai Experimental Animal Center of Chinese Academic of Sciences (Shanghai, China) and were housed in a specific pathogen‐free facility in our hospital. The mice were randomized and injected subcutaneously with 1 × 10^7^ BGC‐823/shCon or BGC‐823/shSTIL cells (n = 10 pre group). The development and growth of implanted tumors were monitored every 4 days up to 3 weeks after inoculation and the tumor volumes were measured with a caliper using the formula of 1/2 × length × Width^2^. The mice were sacrificed and the subcutaneous tumors were dissected and weighed.

### 2.10 Gene microarray and bioinformatics

SGC‐7901/shCon and SGC‐7901/shSTIL cells were harvested and their total RNA was extracted. After quantity and quality of RNA samples using NanoDrop 2000 in an Agilent Bioanalyzer 2100, the transcriptome profiles were assessed with the PrimeView Human Gene Expression Array (Affymetrix, USA), according to the manufacturer's instructions. Briefly, the RNA samples were reversely transcribed into cDNA that were used as the templates, followed by biotin‐labelling using the GeneChip 3'IVT Expression Kit (Affymetrix). The microarray hybridization, washing and staining were performed using GeneChip Hybridization Wash and Stain Kit (Affymetrix). The microarrays were scanned using the GeneChip Scanner 3000 7G to produce raw data. The DEGs were determined with criteria of a *P* < 0.05 or absolute Fold Change > 1. The potential functional pathways of DEGs were analyzed by Ingenuity Pathways Analysis software.

### 2.11 Statistical analysis

Statistical analyses were performed using the SPSS 17.0 and R software. Continuous data are expressed as mean ± SD and analyzed by Student's *t* test for variables normally distributed or *U* test for nonparametric variables. Survival curves were plotted using the R software. The difference was considered statistically significant when a *P* < 0.05.

## 3 RESULTS

### 3.1 Up‐regulated STIL expression is associated with the progression of GC

**Figure 1:**
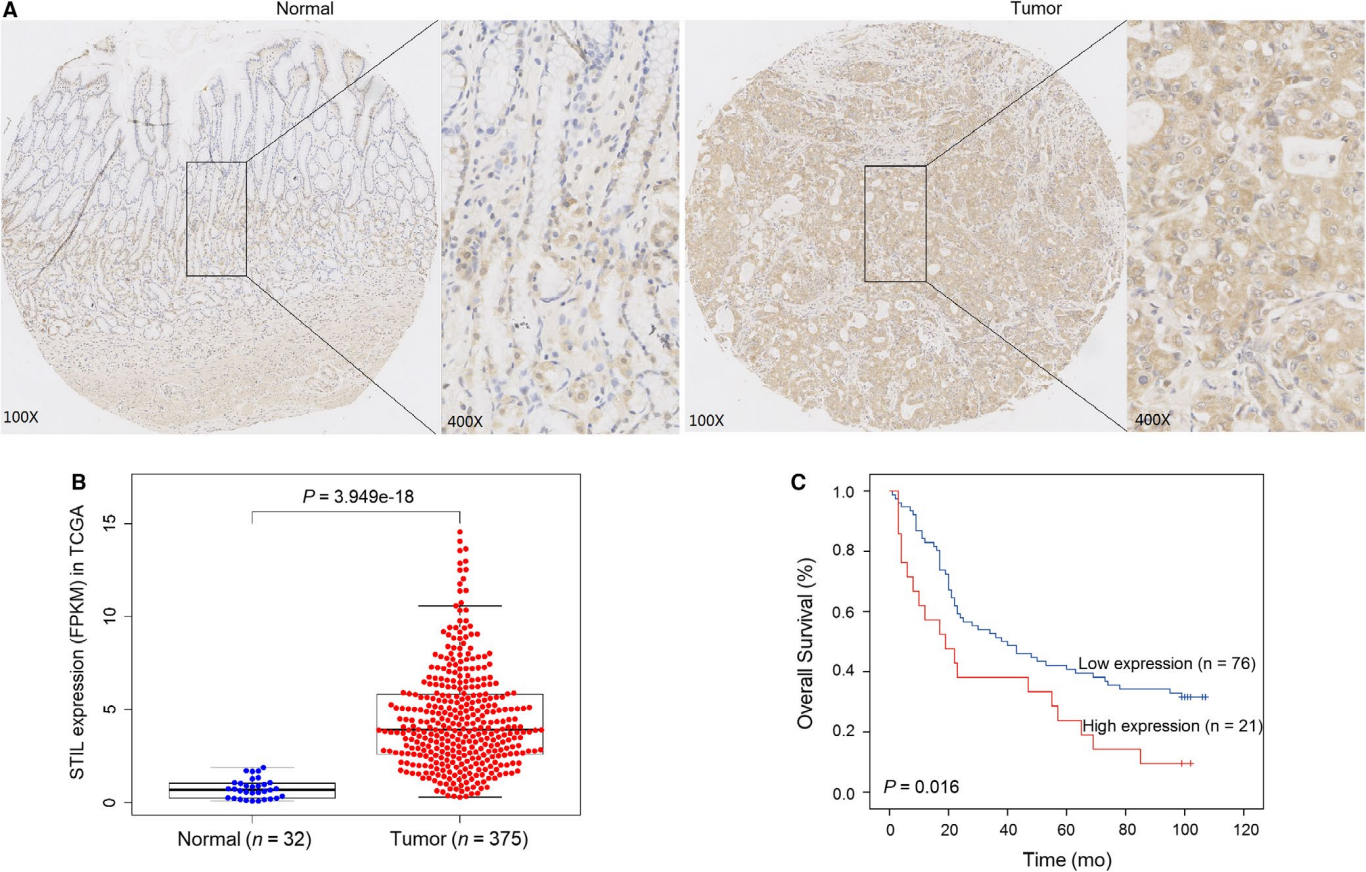
SCL/TAL1 interrupting locus (STIL) expression and its association with overall survival in gastric cancer (GC) patients. (A), Representative micrographs of GC (right) and para‐tumor (left) tissues; (B), STIL mRNA levels in GC and non‐tumor stomach samples in TCGA database; (C), Kaplan‐Meier curves for Overall survival (OS) in GC patients from our cohort

To determine the potential role of STIL in the development of GC, the expression levels of STIL in 100 GC and 80 non‐tumor stomach tissues were determined by TMA‐based immunohistochemistry. As shown in Figure 1A, the STIL expression was up‐regulated in GC compared with that in adjacent non‐tumor tissues. The positivity rate of anti‐STIL staining in GC tissues (63%) was significantly higher than 43.8% in adjacent non‐tumor tissues (*P* = 0.01). Moreover, TCGA data indicated that the relative levels of STIL mRNA transcripts in 375 GC tissues were significantly higher than that in 32 non‐tumor tissues (Figure 1B). In addition, varying levels of STIL mRNA transcripts were detected in the different human GC cell lines (Figure S1). Stratification analysis indicated that the positivity rate of high STIL expression was only significantly associated with T stage of GC in this population (*P* = 0.006), but not with other clinical parameters (Table 1). Further analysis revealed that high STIL expression was a risk factor of poor overall survival in GC patients in this population (Figure 1C, Table 1). Hence, up‐regulated STIL expression was associated with the progression of GC.

**Table 1:** Relationship among clinicopathological variables and SCL/TAL1 interrupting locus (STIL) expression and Overall survival in gastric cancer patients

Variables	STIL expression		*P^a^*	Overall survival (OS)			
				Univariate analysis		Multivariate analysis	
	Low	High		HR (95%CI)	*P* ^b^	HR (95%CI)	*P* ^b^
Gender							
Female	12	24	0.569	1 (Reference)	0.840	NA	NA
Male	25	39		0.95 (059‐1.54)		NA	
Age							
≤65 y	23	27	0.086	1 (Reference)	**0.023**	1 (Reference)	0.061
>65 y	14	34		1.74 (1.08‐2.80)		1.66 (0.98‐2.83)	
Tumor size							
≤5 cm	19	31	0.878	1 (Reference)	**0.002**	1 (Reference)	**0.017**
>5 cm	17	31		2.13 (1.32‐3.42)		1.85 (1.12‐3.08)	
TNM stage							
I+II	4	11	0.371	1 (Reference)	**<0.001**	1 (Reference)	0.055
III+IV	33	52		2.49 (1.50‐4.12)		2.17 (0.98‐4.81)	
T stage							
T1 + T2+T3	34	45	**0.006**	1 (Reference)	**0.005**	1 (Reference)	0.353
T4	2	18		2.13 (1.26‐3.62)		1.36 (0.71‐2.63)	
N stage							
Negative	9	18	0.701	1 (Reference)	**0.037**	1 (Reference)	0.704
Positive	27	45		1.84 (1.04‐3.26)		0.85 (0.36‐2.01)	
M stage							
Negative	35	56	0.453	1 (Reference)	**<0.001**	1 (Reference)	**<0.001**
Positive	2	6		2.49 (1.50‐4.12)		5.24 (2.15‐12.80)	
STIL level							
Low	NA	NA	NA	1 (Reference)	**0.019**	1 (Reference)	0.205
High	NA	NA		1.89 (1.11‐3.20)		1.51 (0.80‐2.83)	

Notes

*P* ^a^ value refers to the association between clinicopathological variables and STIL expression; *P* ^b^ value refers to the association between variables and overall survival. The bold values indicates *P* values < 0.05.

### 3.2 STIL silencing suppresses the proliferation and clonogenicity of GC cells in vitro

**Figure 2:**
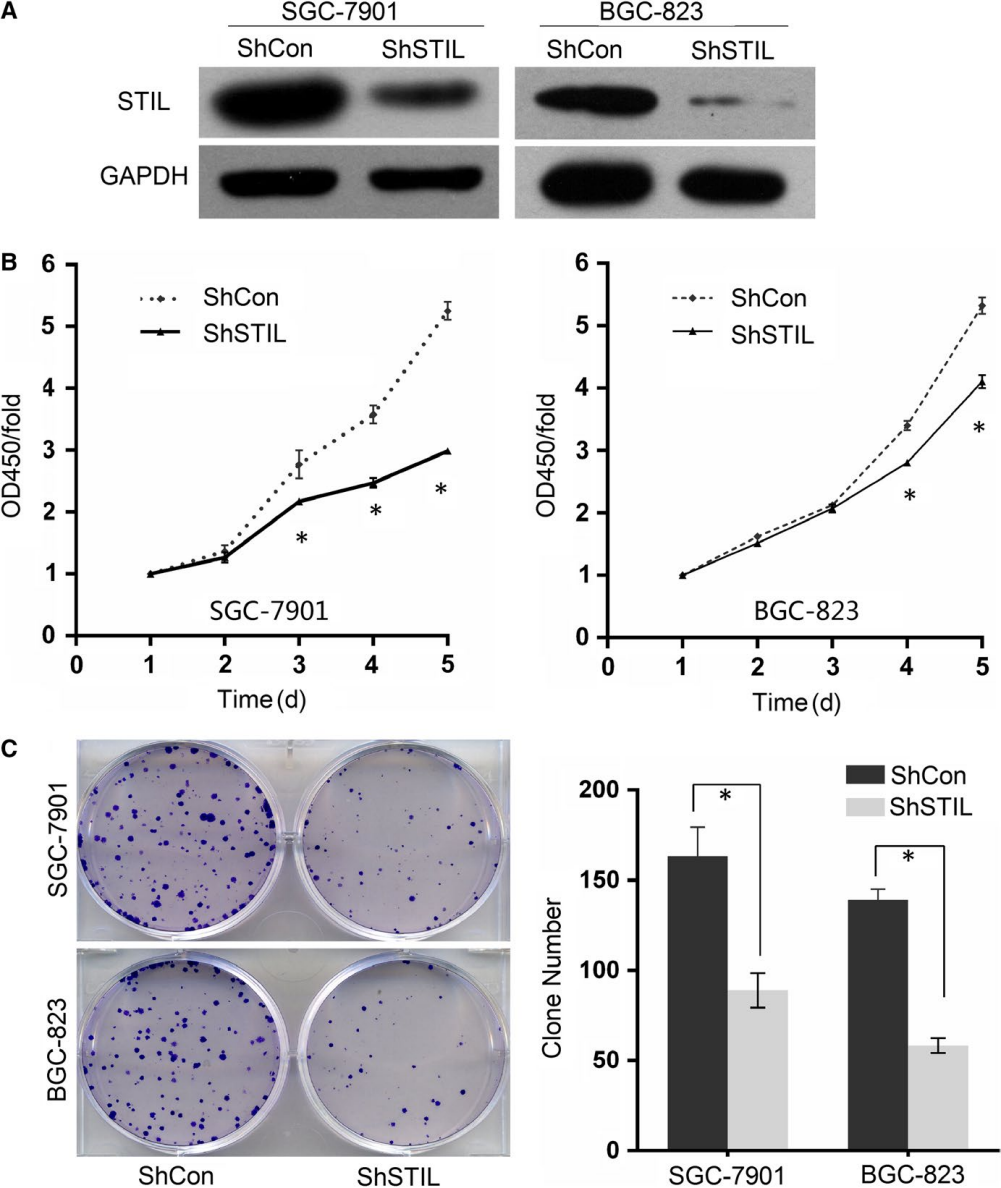
The effect of SCL/TAL1 interrupting locus (STIL) silencing on the proliferation and colony formation of gastric cancer cells. (A), knockdown of STIL in SGC‐7901 and BGC‐823 cells transduced with STIL‐specific and control scrambled shRNAs. (B), knockdown of STIL inhibited cell proliferation determined by CCK‐8. (C), representative micrographs and quantification of crystal violet‐stained cell clones

Next, we employed lentivirus‐mediated STIL‐specific shRNA expression to establish control SGC‐7901/shCon, BGC‐823/shCon and STIL‐silencing SGC‐7901/shSTIL and BGC‐823/shSTIL cells. Western blot analyses indicated that the relative levels of STIL in SGC‐7901/shSTIL and BGC‐823/shSTIL cells were lower than that in SGC‐7901/shCon, BGC‐823/shCon cells (Figure 2A). Subsequently, the impact of STIL silencing on the proliferation and clonogenicity of GC cells were determined. STIL silencing significantly decreased the proliferation of both SGC‐7901/shSTIL and BGC‐823/shSTIL cells (*P* < 0.05, Figure 2B). Similarly, STIL silencing also attenuated the clonogenicity of SGC‐7901/shSTIL and BGC‐823/shSTIL cells (*P* < 0.05 for all, Figure 2C). Collectively, such data indicated that STIL silencing inhibited the proliferation and clonogenicity of GC cells in vitro.

### 3.3 STIL silencing retards the tumor growth in vivo

**Figure 3:**
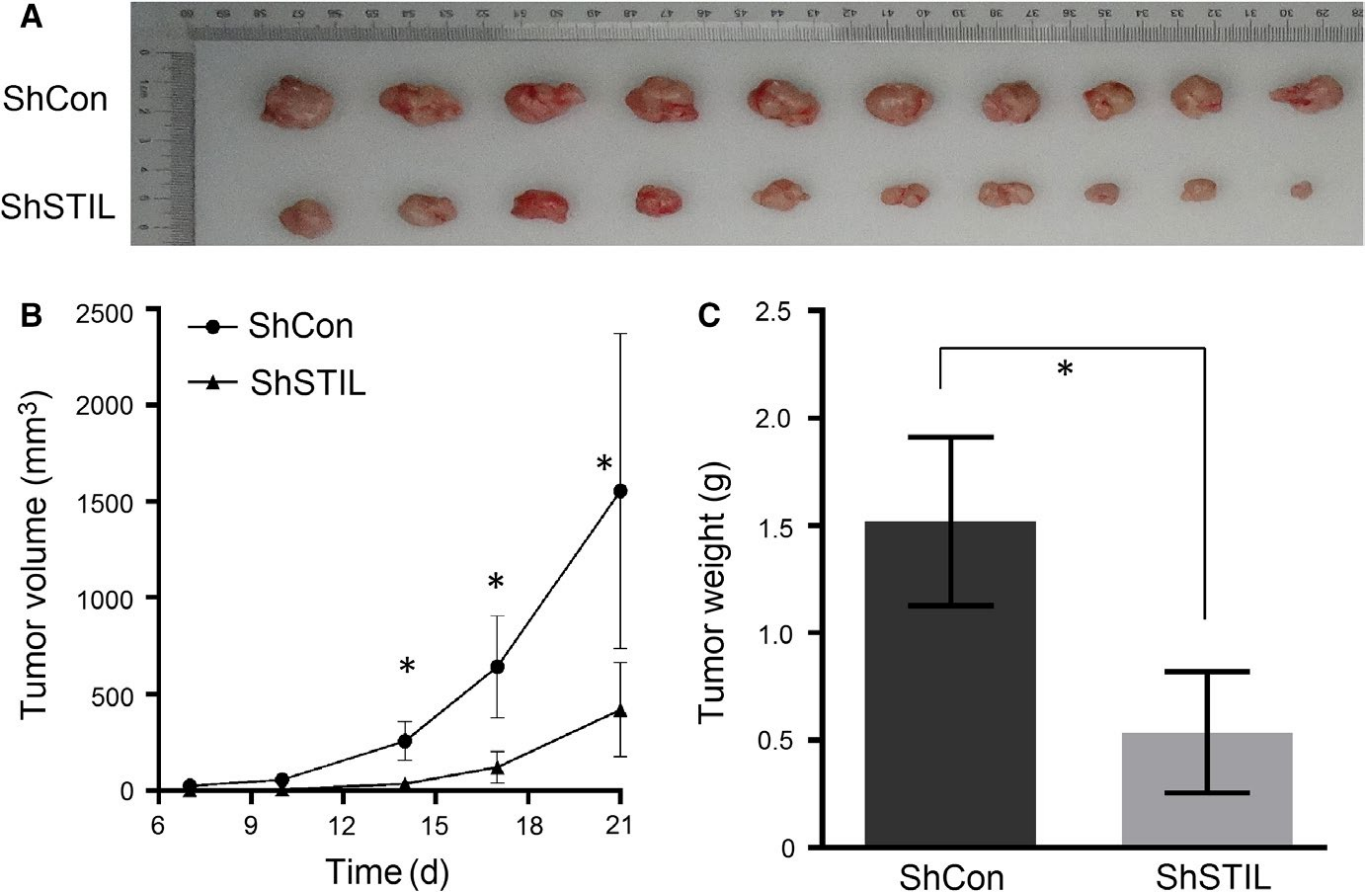
Knockdown of SCL/TAL1 interrupting locus (STIL) retards the development of gastric cancer in vivo. (A), Images of the BGC‐823 tumor xenografts from each mouse (n = 10 per group). (B), Growth curves of tumor xenografts. (C), Tumor weights in sacrificed mice

To further understand the role of STIL in the progression of GC, BALB/c nude mice were implanted with BGC‐823/shCon or BGC‐823/shSTIL and the dynamic growth of implanted tumors was monitored up to 21 days post inoculation. The dissected tumors were photoimaged in Figure 3A and the dynamic growth of BGC‐823/shSTIL tumors was significantly retarded, as compared with that in the BGC‐823/shCon group (*P* < 0.05, Figure 3B). Furthermore, the weights of BGC‐823/shSTIL tumors were significantly less than that of BGC‐823/shCon tumors (*P* < 0.05, Figure 3C). Thus, STIL silencing attenuated the growth of human GC tumors in mice.

### 3.4 STIL silencing induces cell cycle arrest in G2/M phase and apoptosis of GC cells

**Figure 4:**
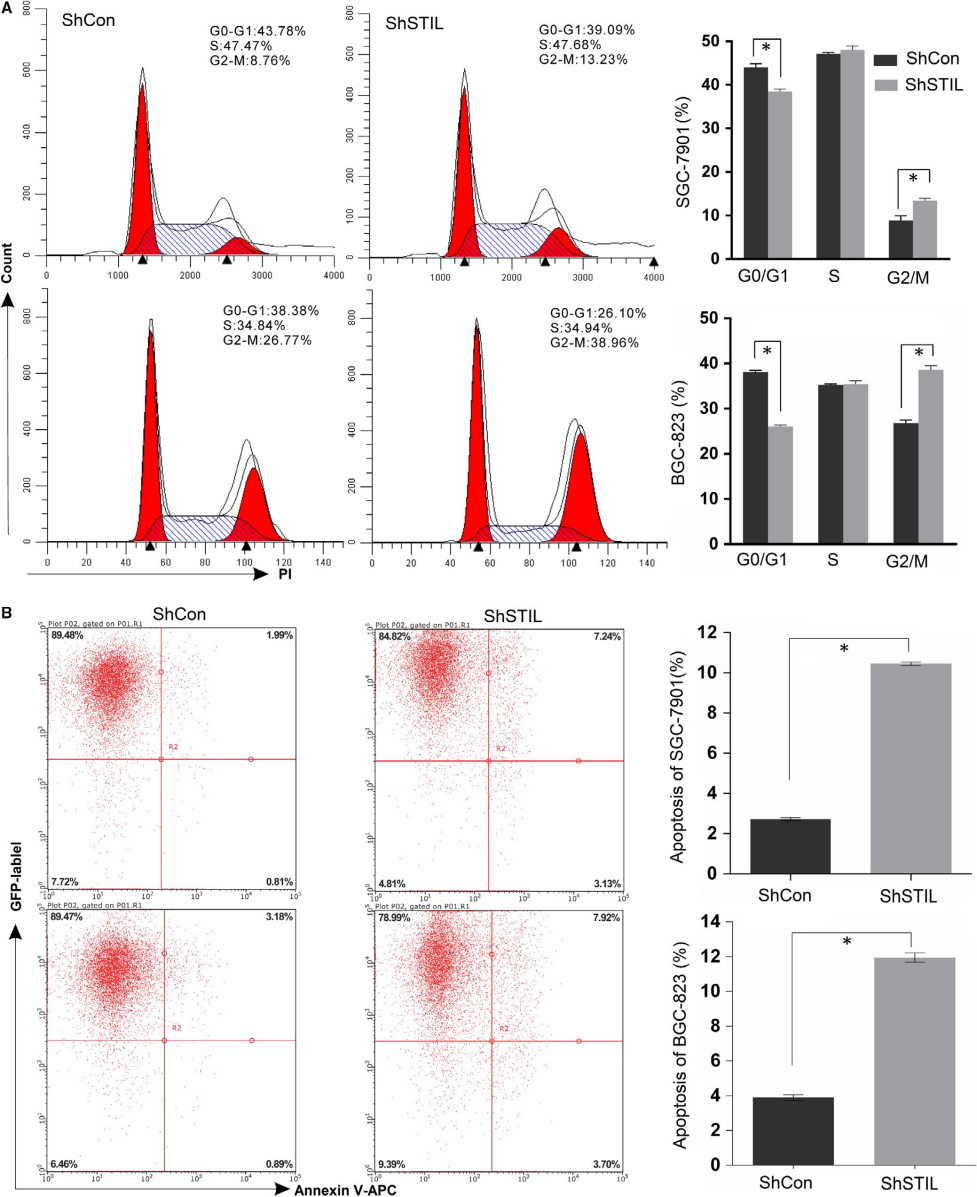
Knockdown of SCL/TAL1 interrupting locus (STIL) induces cell cycle arrest in G2/M and apoptosis of gastric cancer cells. (A), representative images and bar plots of Flow Cytometry analysis in the indicated cells. (B), representative images and bar plots of apoptosis rate in the indicated cells

To understand the mechanisms underlying the action of STIL silencing in inhibiting the proliferation of GC cells, the impact of STIL silencing on cell cycling and apoptosis of GC cells was determined by flow cytometry. As shown in Figure 4A, the frequency of SGC‐7901/shSTIL and BGC‐823/shSTIL cells in G0/G1 phase were significantly lower than that in the control group while the percentages of SGC‐7901/shSTIL and BGC‐823/shSTIL cells in G2/M phase were significantly higher than that in SGC‐7901/shCon and BGC‐823/shCon cells. Further flow cytometry analysis revealed that the percentages of apoptotic SGC‐7901/shSTIL and BGC‐823/shSTIL cells were significantly higher than that of SGC‐7901/shCon and BGC‐823/shCon cells (*P* < 0.05, Figure 4B). Such data indicated that STIL silencing induced cell cycle arrest in G2/M phase and promoted apoptosis of human GC cells.

### 3.5 STIL silencing modulates the global gene expression profiling and signaling in GC cells

**Figure 5:**
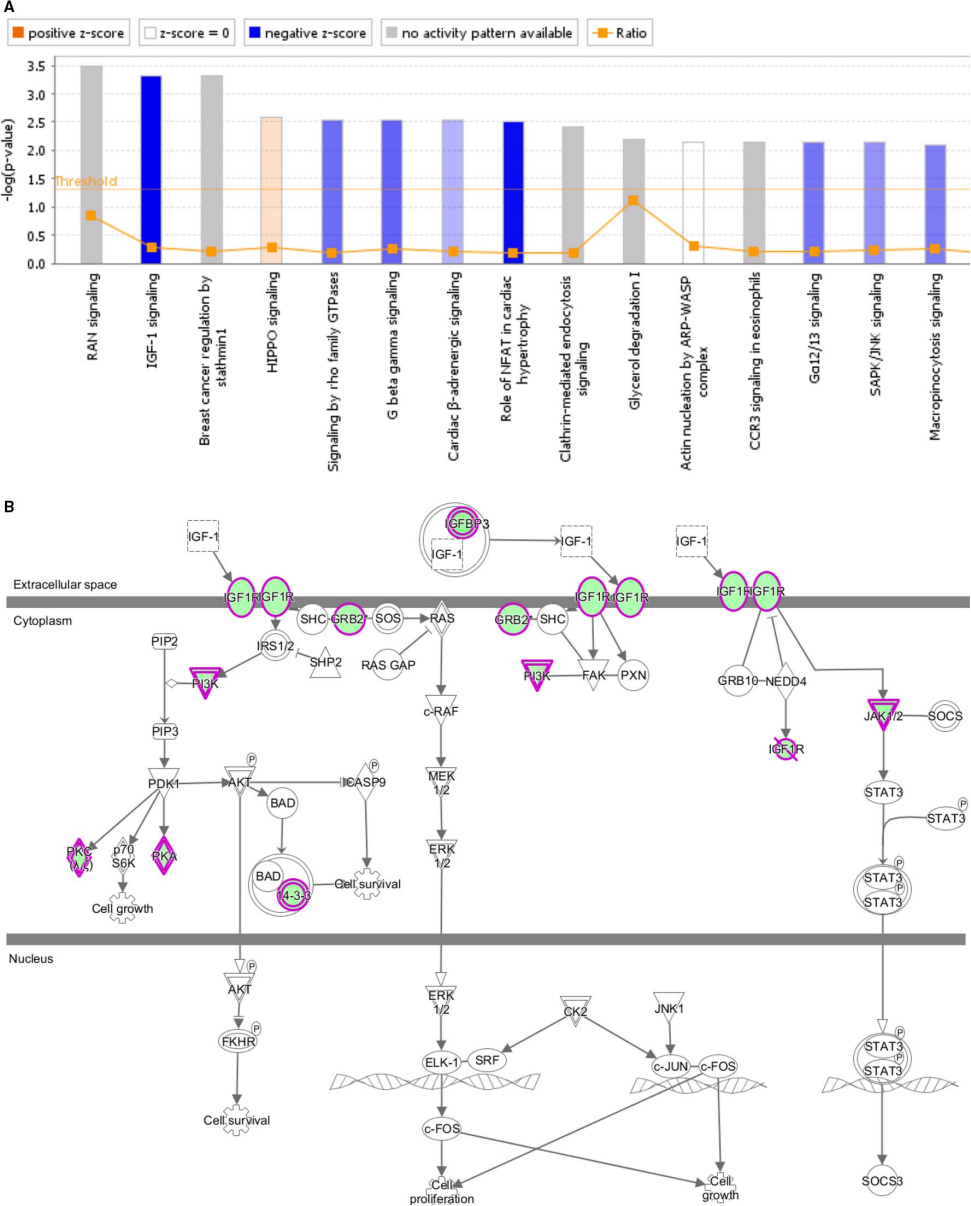
Cluster analysis of differentially expressed genes (DEGs) using Ingenuity Pathways Analysis. (A), DEGs between SGC‐7901/ShSTIL and SGC‐7901/ShCon cells were enriched in 15 pathways. Pathways with “orange” or “blue” color were predicted to be activated (*z*‐score > 0) and inhibited (*z*‐score < 0), respectively. (B), Eight DEGs were enriched in the IGF‐1 pathway. The DEG with “green” color was down‐regulated

**Figure 6:**
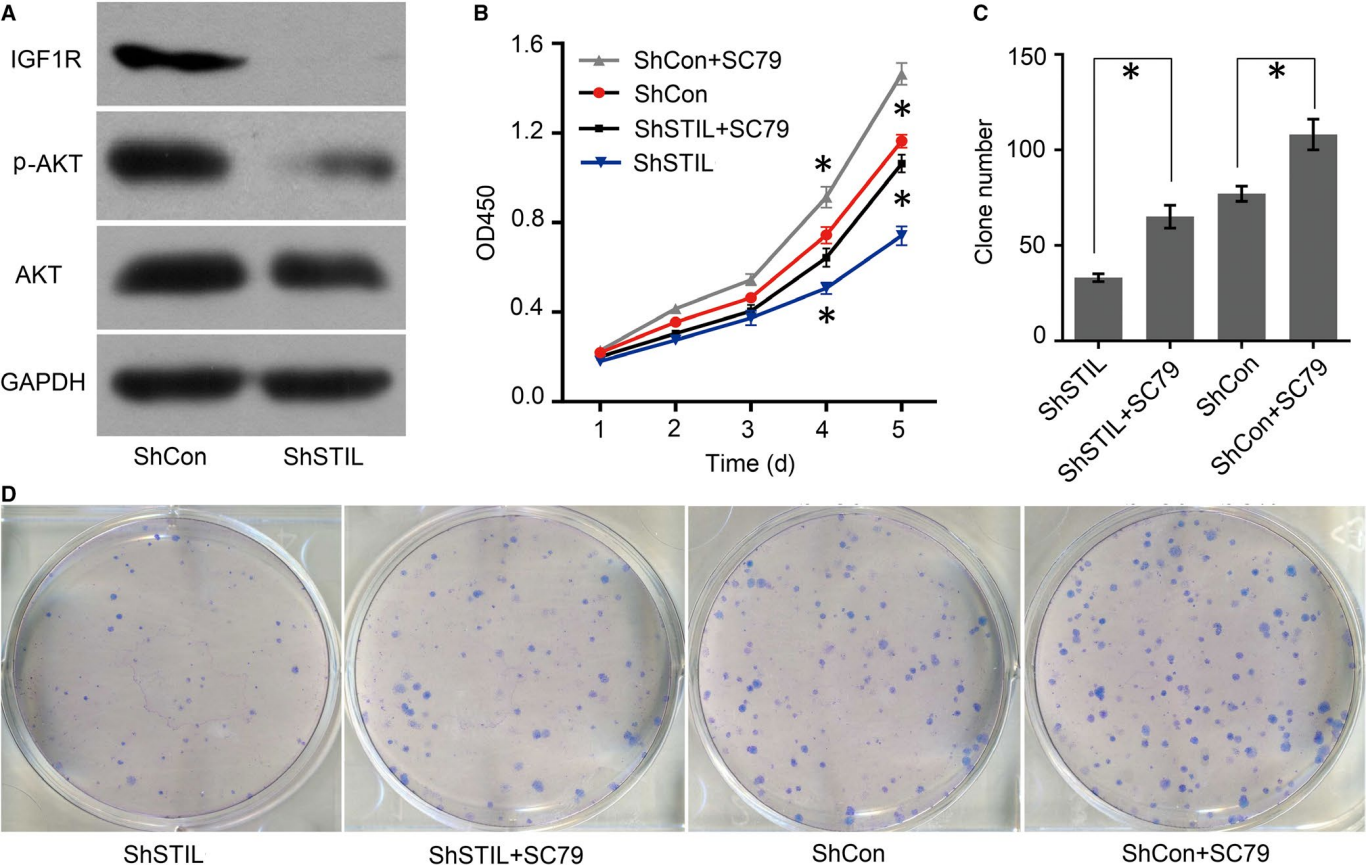
SCL/TAL1 interrupting locus (STIL) promotes gastric cancer (GC) cell proliferation and colony formation through activating the IGF1/PI3K/AKT pathway. (A), Western blot analyses revealed that STIL silencing down‐regulated the IGF1R expression and reduced AKT phosphorylation. (B), CCK8 assay indicated that SC79 treatment promoted the proliferation of GC cells and mitigated the inhibitory effect of STIL silencing. (C, D), SC treatment mitigated the STIL silencing‐decreased clonogencity of GC cells

To gain new insights into the mechanisms by which STIL regulated the progression of GC, the global transcriptome profiles of SGC‐7901/shCon and SGC‐7901/shSTIL cells were compared by microarray. STIL silencing up‐regulated 87 genes and down‐regulated 417 genes in GC cells (Absolute Fold Change > 1, FDR < 0.05). Given that thirty DEGs were controlled by TGF‐β1, the relative levels of their expression were validated by qRT‐PCR. STIL silencing significantly altered the relative levels of 26 out of 30 gene transcripts in SGC‐7901 cells (Figure S2). Bioinformatics analysis indicated that altered genes induced by STIL silencing were markedly enriched in a series of pathways (Figure 5A), and the IGF‐1 signaling ranked the most suppressed one with 9 genes that were down‐regulated, including *YWHAQ, JAK1, PKC, PI3K, GRB2, IGF1R, IGFBP3, PRKAR2A, PRKAG1* (Figure 5B). More importantly, Western Blot revealed that STIL silencing dramatically eliminated or reduced IGF‐1R expression and AKT phosphorylation in SGC‐7901 cells although it did not alter AKT expression (Figure 6A).

### 3.6 Activating the PI3K‐AKT pathway enhances the proliferation and clonogencity of GC cells and mitigated the inhibitory effect of STIL silencing

To identify whether the effect of STIL silencing on GC cells was achieved by attenuating the PI3K/AKT activation, both SGC‐7901/shCon and SGC‐7901/shSTIL cells were treated with SC79, an activator of AKT and their proliferation and clonogenicity were tested. Treatment with SC79 significantly enhanced the proliferation and clonogencity of SGC‐7901/ShCon cells (*P* < 0.05 for both, Figure 6B‐D). Furthermore, treatment with SC79 also significantly increased the proliferation and clonogenicity of SGC‐7901/shSTIL cells. Thus, activating the PI3K/AKT signaling promoted the proliferation and clonogencity of GC cells and mitigated the inhibitory effects of STIL silencing.

## 4 DISCUSSION

SCL/TAL1 interrupting locus has been thought to be an oncogenic factor and its expression is up‐regulated in many types of malignancies. However, there is no report on its role in the progression of GC. In this study, we found that up‐regulated STIL expression was significantly associated with T stage and poor overall survival of GC patients. Knockdown of endogenous STIL inhibited the proliferation and tumorigenicity of GC cells in vitro and in vivo. These findings provide strong evidence that STIL positively regulates gastric carcinogenesis and progression.

Previous studies have shown that STIL mutation causes the left‐right asymmetry defects in mouse,^10^, ^11^ disorganized mitotic spindles in zebrafish^12^ and primary microcephaly in human.^13^ However, elevated STIL expression can trigger chromosome instability and cancer.^14^ Given that STIL is an regulator of cell cycling by promoting the centriolar duplication we tested the impact of STIL silencing on the proliferation and clonogenicity of GC cells. We found that STIL silencing significantly inhibited the proliferation and clonogenicity of GC cells in vitro and the growth of implanted GC in vivo. Furthermore, STIL silencing induced cell cycle arrest in G2/M phase and apoptosis of GC cells. Our findings extended previous observations that STIL silencing hampers the proliferation of colon and cervical cancer cells by inactivating CDK1/Cyclin B1.^15^, ^16^ In contrast, STIL can interact with suppressor‐of‐fused (SUFU) that tethers GLI1 in the cytoplasm while STIL silencing promotes the nuclear accumulation of SUFU and associated GLI1, increasing GLI1‐mediated target gene transcription to promote pancreatic ductal cancer cell proliferation.^8^ Thus, our findings support the notion that STIL exhibits diverse roles through different mechanisms, dependent on the original organ of malignant tumors.

Enrichment analysis of DEGs suggest that STIL silencing may attenuate GC progression through down‐regulating the IGF‐1/PI3K/AKT pathway. Actually, we found that STIL silencing eliminated IGFR1 expression and reduced AKT phosphorylation in GC cells, and treatment with SC79 to activate the PI3K/AKT signaling dramatically mitigated the inhibitory effects of STIL silencing in GC cells. It is well known that the PI3K/AKT signaling is critical for the proliferation, apoptosis, transformation, chemotherapy resistance and other processes in numerous malignancies, including GC.^17^, ^18^ Moreover, the IGF‐1/IGF1R pathway has been reported to be highly activated in the mesenchymal phenotype of GC characterized by a poor prognosis and resistance to chemotherapy compared with the epithelial phenotype.^19^ Given that the IGF‐1/PI3K/AKT pathway is crucial for GC carcinogenesis and progression, STIL‐targeted strategy may be promising for treatment of GC. Nevertheless, the exact mechanisms by which STIL silencing greatly reduced IGF1R expression in GC cells remain elusive and they, together with therapeutic effect of SC79 on the growth of GC in vivo, deserve to be further investigated. Using bioinformatics analysis, we identified SP3 with a zinc finger domain as the transcription factor of *IGF1R* (Table S1). Based on the interactome landscape from BioGRID,^20^ STIL may interact with ubiquitin‐specific protease 7 (USP7),^21^ which represses the auto‐ubiquitination of checkpoint with forkhead and ring finger domains (CHFR)‐E3 ubiquitin ligase.^22^ Intriguingly, SP3 was predicted as a high‐score substrate of CHFR by the UbiBrowser.^23^ Taken together, STIL regulates oncogenic IGF‐1/PI3K/AKT pathway in GC, which may attribute to its interaction with the USP7/CHFR/SP3 signaling axis.

## 5 CONCLUSION

Our data indicated that STIL expression was up‐regulated in GC and associated with the progression of GC. STIL silencing promoted the proliferation and clonogenicity of GC cells by inducing cell cycle arrest and apoptosis, which was achieved by attenuating the IGF‐1/PI3K/AKT pathway. Our findings suggest that STIL may be a novel therapeutic target for intervention of GC and may provide new insights into the regulation of STIL in promoting GC progression.

## CONFLICT OF INTERESTS

The authors declare no conflicts of interest.
