# Supplementary figures and images for: Knockdown of STIL suppresses the progression of gastric cancer by down‐regulating the IGF‐1/PI3K/AKT pathway
Source: J Cell Mol Med. 2019 Jun 11;23(8):5566–75. doi: 10.1111/jcmm.14440 (PMC6653615; doi:10.1111/jcmm.14440)

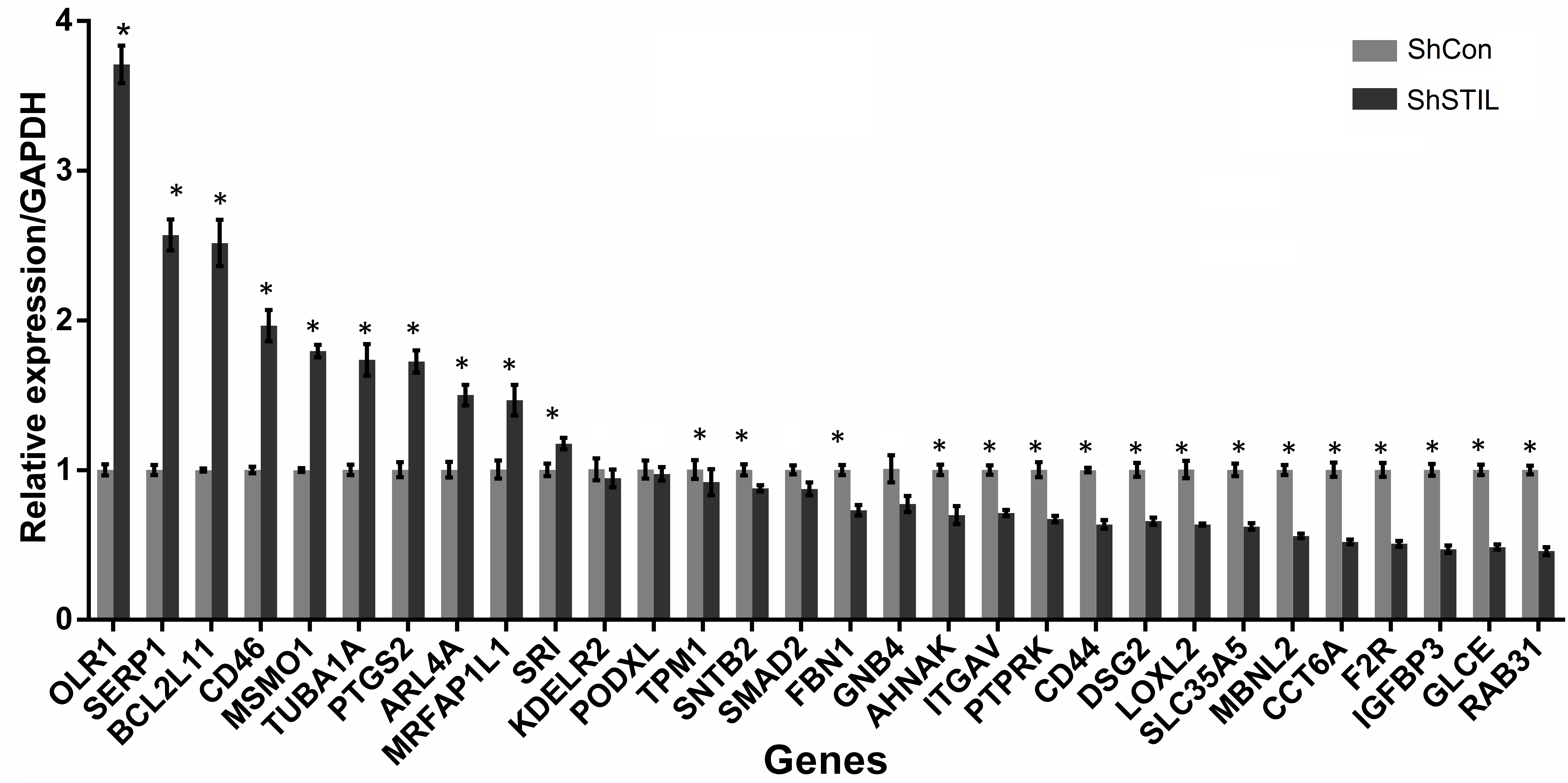

Supplement: Supplementary file 2 [file JCMM-23-5566-s002.tif]
