# Supplementary material for: Knockdown of STIL suppresses the progression of gastric cancer by down‐regulating the IGF‐1/PI3K/AKT pathway
Source: J Cell Mol Med. 2019 Jun 11;23(8):5566–75. doi: 10.1111/jcmm.14440 (PMC6653615; doi:10.1111/jcmm.14440)
Supplement: Supplementary file 3 [file JCMM-23-5566-s003.docx]

**Table S1** Transcription factor binding site (TFBS) prediction of IGF1R

| seqname | TF | start | end | score | strand | squence |
| --- | --- | --- | --- | --- | --- | --- |
| IGF1R\|NM_000875 | SP3 | 579 | 589 | 0.940 | + | TCCCCGCCCAC |
| IGF1R\|NM_000875 | SP3 | 739 | 749 | 0.904 | - | CTCCCGCCCCC |
| IGF1R\|NM_001291858 | SP3 | 579 | 589 | 0.940 | + | TCCCCGCCCAC |
| IGF1R\|NM_001291858 | SP3 | 739 | 749 | 0.904 | - | CTCCCGCCCCC |
